# Prognostic role of plasma vitamin D and its association with disease characteristics in germ cell tumours

**DOI:** 10.3389/fonc.2023.1149432

**Published:** 2023-04-11

**Authors:** Peter Lesko, Barbora Vlkova, Katarina Kalavska, Valentina De Angelis, Vera Novotna, Jana Obertova, Zuzana Orszaghova, Patrik Palacka, Katarina Rejlekova, Zuzana Sycova-Mila, Boris Kollarik, Ramadan Aziri, Daniel Pindak, Jozef Mardiak, Michal Chovanec, Peter Celec, Michal Mego

**Affiliations:** ^1^ 2nd Department of Oncology, Faculty of Medicine, Comenius University and National Cancer Institute, Bratislava, Slovakia; ^2^ Institute of Molecular Biomedicine, Faculty of Medicine, Comenius University, Bratislava, Slovakia; ^3^ Translation Research Unit, Comenius University, National Cancer Institute, Bratislava, Slovakia; ^4^ 1st Department of Oncology, Faculty of Medicine Comenius University (FMCU) and St. Elizabeth Cancer Institute, Bratislava, Slovakia; ^5^ Department of Surgical Oncology, National Institute for Oncology, Bratislava, Slovakia

**Keywords:** cholecalciferol, overall survival, progression-free survival, prognostic biomarker, germ cell tumor

## Abstract

**Background:**

Testicular cancer is the most common malignancy among young men. Vitamin D has pluripotent effects on cancer pathogenesis and plays a role in the metastatic cascade. The aim of this study is to analyze plasma vitamin D in association with clinico-pathological findings and prognosis in patients with germ-cell tumors (GCTs).

**Methods:**

This study included 120 newly diagnosed and/or relapsed GCT patients treated from April 2013 to July 2020, for whom plasma was available in the biobank. Blood samples were drawn the 1st chemotherapy cycle as well as before the 2nd cycle. Plasma vitamin D was measured using ELISA and correlated with disease characteristics and the outcome. For survival analysis, the cohort was dichotomized into “low” and “high” based on median vitamin D.

**Results:**

There was no significant difference in vitamin D plasma levels between healthy donors and GCT patients (p = 0.71). Vitamin D level was not associated with disease characteristics except for brain metastases, where patients with brain metastases had a vitamin D level that was 32% lower compared to patients without brain metastases, p = 0.03. Vitamin D was also associated with response to chemotherapy, with an approximately 32% lower value in patients with an unfavorable response compared to a favorable response, p = 0.02. Moreover, low plasma levels of vitamin D were significantly associated with disease recurrence and inferior progression-free survival (PFS), but not with overall survival (OS) (HR = 3.02, 95% CI 1.36–6.71, p = 0.01 for PFS and HR = 2.06, 95% CI 0.84–5.06, p = 0.14 for OS, respectively).

**Conclusion:**

Our study suggests the prognostic value of pretreatment vitamin D concentrations in GCT patients. Low plasma vitamin D was associated with an unfavorable response to therapy and disease recurrence. However, it remains to be determined whether the biology of the disease confirms a causative role for low vitamin D and whether its supplementation affects the outcome.

## Introduction

Testicular cancer is the most common cancer among young men below 34 years old; however, its overall contribution to all cancers among men is only 1% (1). Cure rates reach more than 90% (2), mostly depending on the clinical stage of the disease and associated patient symptoms (3). However, GCTs represent a heterogeneous group of patients, and the prognosis substantially varies according to the International Germ Cell Collaborative Group (IGCCCG) classification, with 5-year survival ranging from 98% in the good-risk group to 50%–70% in the poor-risk group (4, 5). Current prognostic biomarkers widely used are encompassed in the IGCCCG risk stratification model, such as serum tumor markers and extension of disease according to tumor, node, and metastasis status (6). Despite this, new biomarkers with further proper prognostic stratification in germ cell populations are still needed. Programmed death ligand-1 status as well as neutrophil–lymphocyte ratio or systemic inflammatory index were evaluated as prognostic biomarkers in several studies (7, 8), with favorable results as independent predictors of outcome in a selected population of germ cell tumors, but their usefulness in practice has not been established. Thus, understanding disease better with more precise identification of patients with an adverse prognosis is crucial to improving patient management, as might be disease monitoring.

Vitamin D is the precursor of the steroidal hormone calcitriol (9), which is currently investigated among various cancers such as gastrointestinal cancers, breast cancers, and urological cancers (10, 11). It is generally known that vitamin D plays its key role in the regulation of calcium metabolism (12); moreover, vitamin D is an inducer of cell differentiation and promotes inhibition of epithelial–mesenchymal transmission (13, 14); as well, vitamin D shares antiangiogenic properties (15, 16), regulates autophagy (17, 18), and induces apoptosis (19, 20). Vitamin D mediates its function *via* the vitamin D receptor (21), which is being expressed on immune cells (22), as well as in germ cell tumor tissue, unlike tumor-free testicular tissue (23). Therefore, immunity regulation is also affected by vitamin D in terms of shifting T-cell maturation from the Th1 to the Th2 phenotype as well as the polarization of tumor-associated macrophages from the M1 to the M2 type, where antitumor abilities are promoted (22). Moreover, inhibition of differentiation and mutation of dendritic cells, along with inhibition of B-cell proliferation, is caused by vitamin D (24). To conclude, suppression of pro-inflammatory cytokines and increased production of anti-inflammatory cytokines are mediated by vitamin D (24). The role of vitamin D in testicular germ cell tumors (TGCTs) was assessed in this study (23); a significant anti-proliferative effect on testicular germ cell tumor lines was found. Along with that, vitamin D also affects gene expression in TGCT lines, such as upregulation tumor suppressor genes along with upregulation of MAP kinase-activated protein kinase 2 (MAPKAP2) (23), which may play a role in cancer invasiveness (25). Moreover, differentiation of GCT is also affected by vitamin D. Downregulation of pluripotency genes as well as inducement of mesenchymal transmission toward an osteogenic phenotype in embryonal carcinoma cells were observed (26). Therefore, it seems that vitamin D may operate as an inhibitor of cell proliferation and most likely have an impact on metastasis. Thus, the question arises whether vitamin D might be associated with more favorable tumor features as well as with tumor behavior such as less aggressive behavior.

The prognostic association of vitamin D serum levels was also studied (27–29). Studies have shown that low plasma levels of vitamin D in patients are seen in advanced stages of disease (30, 31). Moreover, a prognostic association between plasma levels of vitamin D and overall and/or progression-free survival was evaluated in cancers such as melanoma, colorectal, breast, and Hodgkin´s lymphoma (32–34), where a positive correlation between vitamin D plasma value with progression-free survival (PFS) and/or overall survival (OS) was established. Due to this date, there was no specific study that addressed the prognostic significance of vitamin D in testicular germ cell tumors.

Studies evaluating the function of vitamin D proposed that the metastatic cascade is affected by the vitamin D plasma serum level (35–38); therefore, we hypothesize that there might be an association between the site and/or presence of metastases and the vitamin D plasma level in germ cell tumor patients. Positive association between low vitamin D level and promotion of bone metastasis as well as lung metastases in mice with breast tumor models was well described (36, 39); thus, potential correlation between clinic-pathological variables in testicular germ cell tumor and vitamin D plasma level might be present as well.

The aim of this study was to assess the relationship between clinico-pathological characteristics and vitamin D plasma levels in patients with GCT, as well as to determine its impact on prognosis in specific subpopulations.

## Methods

### Study population

The study included 120 newly diagnosed and/or relapsed GCT patients treated with first-line or salvage chemotherapy from April 2013 to July 2020 at the National Cancer Institute and/or St. Elizabeth Cancer Institute, for whom plasma was available in the biobank. Vitamin D supplementation in the studied group was not assessed in this study. Data regarding tumor histology, chemotherapy regimens, stage of disease, type and site of metastasis, and other patient and tumor characteristics were recorded and correlated with vitamin D levels. The study was approved by the institutional review board of the National Cancer Institute of Slovakia. The study included 21 age-matched healthy donors (HD) as well. Each participant, including healthy donors, signed informed consent before the study was initiated.

### Plasma sample collection

From each enrolled study subject, an atraumatic peripheral blood sample of 1 ml was collected at the antecubital fossa and transferred into EDTA-treated collection tubes (BD Vacutainer^®^) at baseline in the morning on day -1 or 0 of the first or later line (>1) of chemotherapy. Collected blood samples were centrifuged at 5,000 rpm for 10 min at room temperature within 2 h of venipuncture. Obtaining supernatants were aliquoted into 1-ml aliquots that were archived at −80°C until further analysis.

### Vitamin D

A commercial ELISA kit was used for the quantification of 25-hydroxyvitamin D (catalog number DE1971, Demeditec Diagnostics, Kiel, Germany). With an analytical sensitivity of 3 ng/ml, the kit was chosen for the expected low concentrations in the collected samples. Technical variability expressed as intra- and inter-assay coefficients of variation was 3% and 10%, respectively.

### Determination of leukocyte immunophenotypes

Leukocyte immunophenotypes were determined in peripheral blood samples collected from analyzed GCT patients in an EDTA-treated collection tube. Tested samples were processed within 24 h following collection, as described below.

Briefly, leukocytes were stained using fluorochrome-conjugated antibodies from BD Pharmingen, and subsequently, leukocytes with defined immunophenotypes were quantified using flow cytometry (Canto II Cytometer; Becton, Dickinson and Company, Franklin Lakes, NJ, USA). The antibody combinations used for the basic panel, the regulatory T-cell panel, the dendritic-cell (DC) panel, and the myeloid-derived suppressor-cell panel were used in the same scheme as that described by Kalavska et al. (40). Used antibodies are also listed in the supplementary material. A cocktail of applied antibodies was incubated with 300,000–500,000 white blood cells in 200 µl for 20 min at room temperature. For the assessment with a BD FACSCanto™ II flow cytometer (Becton Dickinson, Franklin Lakes, NJ, USA), a minimum of 100,000 leukocytes were utilized. KALUZA software (Beckman Coulter, Inc., Brea, CA, USA) was used for the analysis of the flow cytometry data. Forward scatter (FSC) and side scatter were used to exclude debris according to size and granularity, while the exclusion of doublets was performed using FSC-Height and FSC-Area. The number of gated cells considered the minimum for evaluation was 100.

### Statistical analysis

The data were tabulated. Characteristics were summarized using the mean or median (range) for continuous variables and the frequency (percentage) for categorical variables, respectively. Statistical analysis was performed using non-parametric tests as the distribution of vitamin D expression was with non-normal distribution (Shapiro–Wilk test). The non-parametric Kruskal–Wallis test was used for the analysis of the association of serum vitamin D expression with clinico-pathological variables between two and more than two groups of patients. The independence of two group variables with dichotomized vitamin D levels (“low” vs “high” based on median value) was compared by the Fischer test. PFS was calculated from the date of starting treatment with chemotherapy to the date of progression, death, or the date of the last adequate follow-up. OS was calculated from the date of starting chemotherapy treatment to the date of death or last follow-up. A univariate Kaplan–Meier statistical approach was used to assess the outcome of survival data in conjunction with vitamin D status (“high,” defined as above the median vs. “low,” below the median) in certain populations among our studied group. Moreover, vitamin D plasma levels, which were available before the 1st and 2nd cycles of chemotherapy (CTx) in the same patients, were stratified into four groups based on the dichotomized value of vitamin D (Low-Low (LL), Low-High (LH), High-Low (HL), and High-High (HH)) and compared in univariate Kaplan–Meier models for PFS and OS as well. All p-values presented are two-sided, and associations were considered significant if the p-value was less than or equal to 0.05. Statistical analyses were performed with NCSS 2022 statistical software.

## Results

### Patients’ characteristics

From April 2013 to January 2020, the study encompasses 120 patients with diagnosed GCTs and 21 age-matched healthy donors. Most of the patients were treatment naïve 105 (90%), while 12 (10%) were treated with salvage chemotherapy. The median age of the studied population was 34 years, compared to a median age of 35 years for healthy donors (p = 1.00). **Table 1** summarizes patient and/or disease characteristics. The majority of patients had non-seminoma histology, prevailing at clinical stage III, and were mostly represented as having a low-risk disease, according to the IGCCCG classification. The most common site of metastases was the retroperitoneum, followed by the lungs, while brain metastases were revealed in only six patients.

**Table 1:** Patients’ characteristics.

Patient´s characteristics	N	%
Histology		
Seminoma	33	27.50
non-seminoma	87	72.50
Primary site		
Testis	114	95.00
Extragonadal	5	4.20
NA	1	0.8
Chemotherapy line		
1st line	105	90
>1st line	12	10
NA	3	2.5
Response to chemotherapy		
Complete response	57	47.50
Partial response marker negative	41	34.17
Partial response marker positive	5	4.17
Stable disease	1	0.83
Progressive disease	8	6.67
Favorable response	99	82.5
Unfavorable response	16	13.33
NA	5	4.17
Progression free survival		
Alive	90	75
Relapse	24	20
NA	6	
Overall survival		
Alive	95	79.17
Death	20	16.67
NA	5	4.16
Stage of disease		
I.A stage	2	1.67
I.B stage	11	9.17
I.S stage	5	4.17
II.A stage	12	10.00
II.B stage	21	17.50
II.C stage	11	9.17
III.A stage	9	7.50
III.B stage	14	11.67
III.C stage	25	20.83
Relapsed disease	9	7.5
NA	1	0.83
Serum tumor marker		
S0	36	30.00
S1	41	34.17
*S2*	20	16.67
S3	22	18.33
NA	1	0.83
IGCCCG classification		
Good	75	62.50
Intermediate	11	9.17
Poor	23	19.17
NA	11	9.16
Metastatic site		
Retroperitoneal Lymphadenopathy	98	81.67
Mediastinal lymphadenopathy	17	14.17
Lung metastases	32	26.67
Pulmonary metastases + lymph node except retropertoneum (M1a)	41	34.17
Brain metastases	6	5.00
Liver metastases	17	14.17
another site of metastases	8	6.67
Visceral non-pulmonary metastases (M1b)	19	15.83
N0	20	16.67
N1	16	13.33
N2	33	27.50
N3	49	40.83
another site of lymphadenopathy	19	15.83
Number of metastatic sites		
0	19	15.83
1–2	69	57.5
≥3	27	22.5
NA	5	4.17

N, number; S, stage; mts, metastatis; N1–3, Lymphadenopathy (sizes 1–3); IGCCCG, International Germ Cell Collaborative Cancer Group; NA, not available.

### Comparison of vitamin D plasma level between GCT and healthy donors

In our studied group, we did not observe a significant difference in vitamin D plasma level between healthy donors and GCT patients (mean ± standard error of the mean was 15.7 ± 1.6 ng/ml vs. 15.9 ± 0.7 ng/ml, p = 0.71). The vitamin D plasma level of healthy donors was also compared to the vitamin D level in GCT patients in a month-matched manner to exclude differences due to sun exposure; no significant difference was observed (p = 0.23).

### Association between mean plasma vitamin D level and patients and/or tumor characteristics

The mean plasma level of vitamin D ± SEM was compared between groups of patients with different clinico-pathological variables (**Table 2**). There was no difference in plasma level and patient and/or tumor characteristics except for the number of metastatic sites (>3) and brain metastases, which were inversely associated with vitamin D level. A significant difference was also retained when comparing healthy donors to GCT patients with brain metastases (10.9 ± 2.4 ng/ml vs 15.7 ± 1.3 ng/ml, p = 0.03); however, the number of patients with brain metastases was only six. We were not able to confirm similar results in GCT patients with metastatic sites (>3) (p = 0.13). We also compared vitamin D values among GCT patients in the 1st-line chemotherapy setting to patients with relapse, where a significant difference in vitamin D value was not observed (p = 0.06).

**Table 2:** Vitamin D associations patients/tumor characteristics.

	Vitamin D							Low		high		
Variable	N	Mean (ng/ml)	Median (ng/ml)	SD (ng/ml)	Q1 (ng/ml)	Q3 (ng/ml)	P-value	N	%	N	%	P-value
Histology												
Seminoma GCT	33	15.9	13.5	7.9	9.7	20.5	0.92	16	48.5	17	51.5	1.00
non seminoma GCT	87	15.9	13.3	7.6	11.3	17.8		44	50.6	43	49.4	
Primary site												
Testis site	114	15.7	13.2	7.6	10.9	17.6	0.30	59	51.8	55	48.2	0.21
Extragonadal	5	16.6	16.1	4.2	13.7	20.1		1	20	4	80	
Chemotherapy line												
1st line	105	16.2	13.6	7.8	11.1	20	0.07	50	47.6	55	52.4	0.13
>1st line	12	11.9	12.6	2.2	9.9	13.5		9	75	3	25	
Stage of disease												
I.A–I.B stage	13	12.9	12.3	3.2	11.1	15.8	0.75	7	53.9	6	46.1	0.72
I.S stage	6	16.5	12.9	7.4	12.8	22.4		4	66.7	2	33.3	
II.A–III.A stage	52	16.5	13.7	8.7	10.5	20.2		25	48.1	27	51.9	
III.B–III.C stage	39	16.2	13.8	7.6	11.3	20.2		18	46.2	21	53.8	
Relapsed	9	12.58	13.2	1.97	11.7	13.5		6	66.7	3	33.3	
Serum tumor marker												
S0	36	15.2	12.7	8.2	10.0	19.5	0.36	20	55.6	16	44.4	0.23
S1	41	15.5	13.1	7.3	10.8	16.4		23	56.1	18	43.9	
S2	20	18.3	15.7	8.2	11.4	23.4		6	30.0	14	70.0	
*S3*	22	14.7	13.3	5.9	11.7	15.5		11	50.0	11	50.0	
IGCCCG class.												
good	75	16.1	13.4	8.1	10.8	13.4	0.82	37	49.3	38	50.7	0.93
intermediate	11	16.4	13.9	7.4	11	21.5		5	45.5	6	54.6	
poor	23	15.4	12.7	7.1	11.4	15.9		12	52.2	11	47.8	
Metastatic sites												
Retroperitoneal metastases												
Present	98	16.3	13.7	7.9	11	20.2	0.15	46	46.9	52	53.1	0.15
absent	21	13.3	12.5	4.4	11.3	14.9		14	66.7	7	33.3	
Medistinal Lymphadenopathy												
Present	17	13.4	11.4	5.3	10.5	14.8	0.07	12	70.6	5	29.4	0.11
Absent	102	16.1	13.6	7.8	11.4	18.9		48	47.1	54	52.9	
Pulmonary metastases												
Present	32	14.2	13	6.0	11.1	15.7	0.27	19	59.4	13	40.6	0.32
absent	87	16.3	13.6	7.9	10.9	20.2		41	47.1	46	52.9	
Pulmonary metastases + lymph node except RP (M1a)												
Present	41	17.5	13.9	9.5	11.5	21	0.39	19	46.3	22	53.6	0.70
absent	69	15.1	13.1	6.4	10.8	17.9		35	50.7	34	49.3	
Brain metastases												
Present	6	10.9	11.6	1.9	8.6	12.2	0.03	6	100	0	0.00	0.03
Absent	113	16.0	13.5	7.6	11.2	19.2		54	47.8	59	52.2	
Liver metastases												
Present	17	14.9	13.1	6.0	11.3	18.0	0.72	10	58.8	7	41.2	0.60
Absent	102	15.9	13.5	7.8	11.0	18.0		50	49.0	52	51.0	
Another site of metastases												
Present	8	10.4	10.0	2.2	8.6	12.7	0.004	7	87.5	1	12.5	0.06
Absent	111	16.1	13.6	7.6	11.3	19.9		53	47.8	58	52.3	
Non-pulmonary visceral metastases (M1b)												
Present	19	14.3	12.1	5.9	10.5	15.7	0.39	12	63.2	7	36.8	0.31
absent	93	16.3	13.6	8.0	11.2	20.0		44	47.3	49	52.7	
Lymphnode status												
N0-1	36	15.4	13.1	8.0	11.0	16.3	0.71	19	52.8	17	47.2	0.84
>N1	82	15.9	13.5	7.4	11.0	20.1		40	48.9	42	51.1	
Another Lymphadenopathy												
Present	19	15.6	13.2	7.0	12	20.1	0.99	11	57.9	8	42.1	0.62
Absent	100	15.8	13.5	7.6	10.9	18.0		49	49.0	51	51.0	
Number of metastatic sites												
0	19	13.5	12.8	4.6	11.4	15.5	0.04	12	63.2	7	36.8	0.002
1–2	69	17.4	15.3	8.7	11.1	21.1		26	37.7	43	62.3	
≥3	27	13.5	12.1	4.8	10.9	13.5		20	74.1	7	25.9	
The best Response to Ctx												
CR + PRm-	98	16.3	13.7	7.9	11.3	20.0	0.03	46	46.9	53	53.1	0.01
PR marker +	5	10.4	10.9	1.8	8.7	12.0		5	100	0	0.00	
Stable Disease	1	7.5	7.5	NA	7.5	7.5		1	100	0	0.00	
Progressive disease	8	13.1	13.1	2.1	12.0	15.1		6	75.0	2	25.0	
Unfavorable response												
Present	16	11.2	11.7	2.2	8.9	13.1	0.02	14	87.5	2	12.5	0.002
Absent	99	16.5	13.9	7.9	11.3	20.2		44	44.4	55	55.6	
PFS												
without relapse	90	16.1	13.8	8.0	11.3	18	0.17	40	44.4	50	55.6	0.01
relapse	24	13.7	12,3	5,2	10.6	14.9		18	75.0	6	25.0	
Overall survival												
alive	95	16.0	13.7	7.9	11.2	18.9	0.32	44	46.3	51	53.7	0.08
death	20	14.0	12.8	5.4	10.6	15.3		14	70.0	6	30.0	

SD, standard deviation; TGCT, testicular germ cell tumor; CTx, chemotherapy; S, stage; IGCCCG, International Germ Cell Collaborative Cancer Group; PFS, progression free survival; vit., vitamin; PRm+, partial response marker positive; CR, complete response; PRm−, partial response marker negative; GCT, germ cell tumor; Q, quartile.

There was a significant difference in the mean vitamin D level depending on response to the chemotherapy regimen. High levels of vitamin D were observed in patients with complete responses and partial response markers negative compared to patient in whom partial response markers were positive stable or progressive disease was stated (p = 0.03). Similarly, a significantly lower mean vitamin D value was associated with patients in whom an unfavorable response (other than complete remission and/or partial remission with negative serum tumor markers) was experienced (p = 0.02). Moreover, a significant difference in vitamin D level was observed when poor responders (patients with an unfavorable response to chemotherapy) were compared to healthy donors (10.8 ng/ml vs 15.7 ng/ml, p = 0.0002). The vitamin D plasma value was also compared before the 1st cycle of chemotherapy and again after the 2nd cycle among all studied patients; however, this difference was not statistically significant.

### Association between vitamin D plasma level and specific immune cell subpopulations

Statistical analysis of a possible link between the level of vitamin D in the plasma of GCT patients and changes in the percentage of evaluated innate immune cells revealed that the level of vitamin D in the plasma positively correlated with the number of eosinophils (p = 0.009) and the percentage of CD4-positive NKT cells, (p = 0.02). On the other hand, an inverse association was determined between plasma vitamin D and CD16+ HLADR+ Lin− dendritic cells (DCs) (p = 0.01).

Assessing selected adaptive immune cells subpopulations, we found no significant link between value of vitamin D in plasma of GCT patients and adaptive immune cells percentage.

### Prognostic value of vitamin D level and patients’ outcome

The median follow-up of all patients was 22.6 months (ranging from 0.1 to 100.4 months); 24 patients (20%) experienced disease relapse, and 19 patients (16.67%) experienced death. Patients who experienced disease relapse had a lower vitamin D level compared to patients without recurrence (13.7 ± 1.5 ng/ml vs 16.1 ± 0.8 ng/ml, p = 0.01).

**Figure 1:**
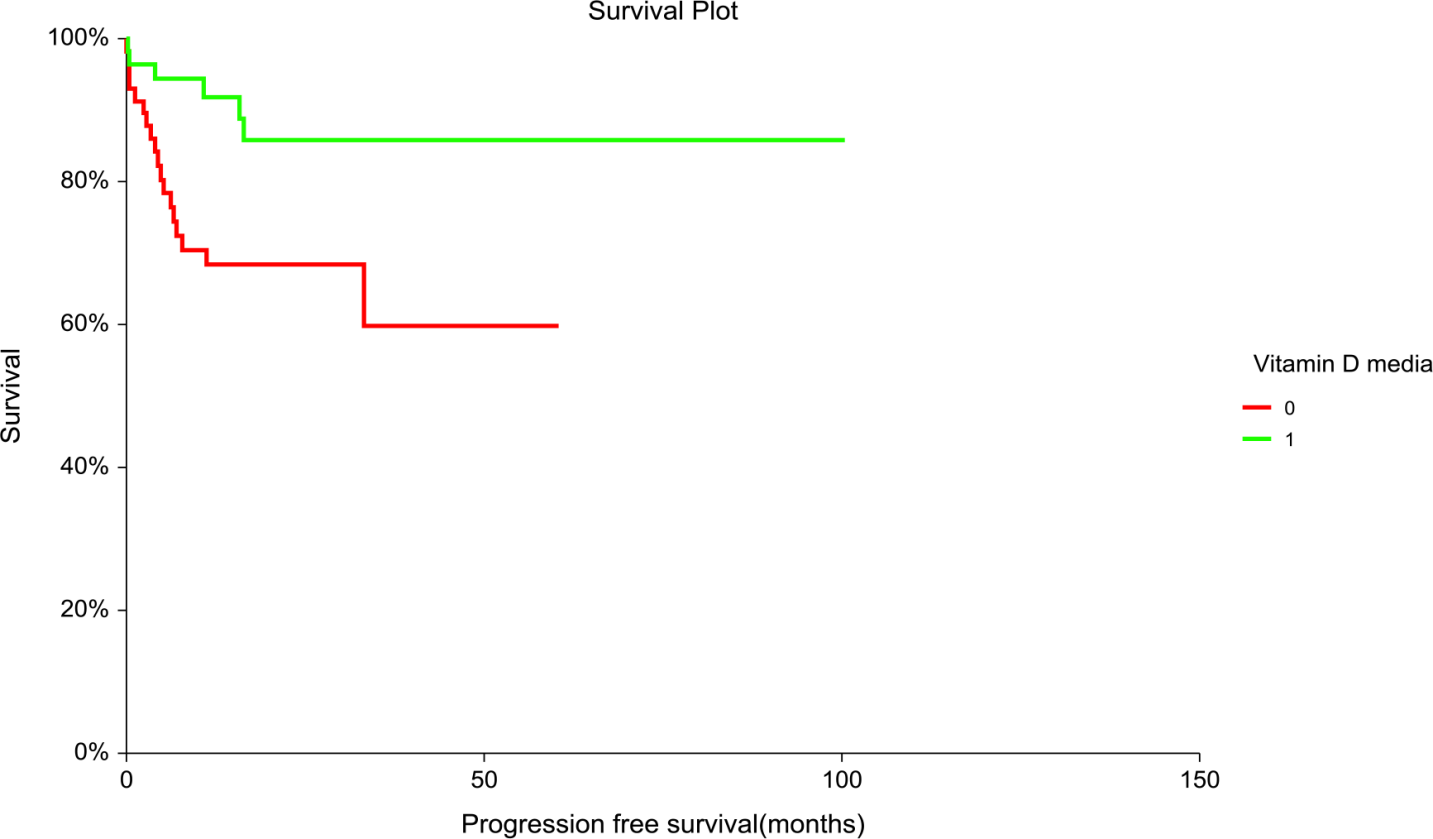
Kaplan–Meier estimates of probabilities of progression-free survival according to plasma vitamin D concentration in TGCT patients (N = 120). Patients with plasma vitamin D concentration above median had significantly better PFS as compared to patients with lower vitamin D concentration [HR] = 3.02, 95% CI (1.36–6.71), p = 0.014; 0 = plasma vitamin D concentration below median, 1 = plasma vitamin D concentration above median). HR, hazard ratio; TCGT, testicular germ cell tumor; PFS, progression free survival; 0, plasma vitamin D concentration below median; 1, plasma vitamin D concentration above median.

**Figure 2:**
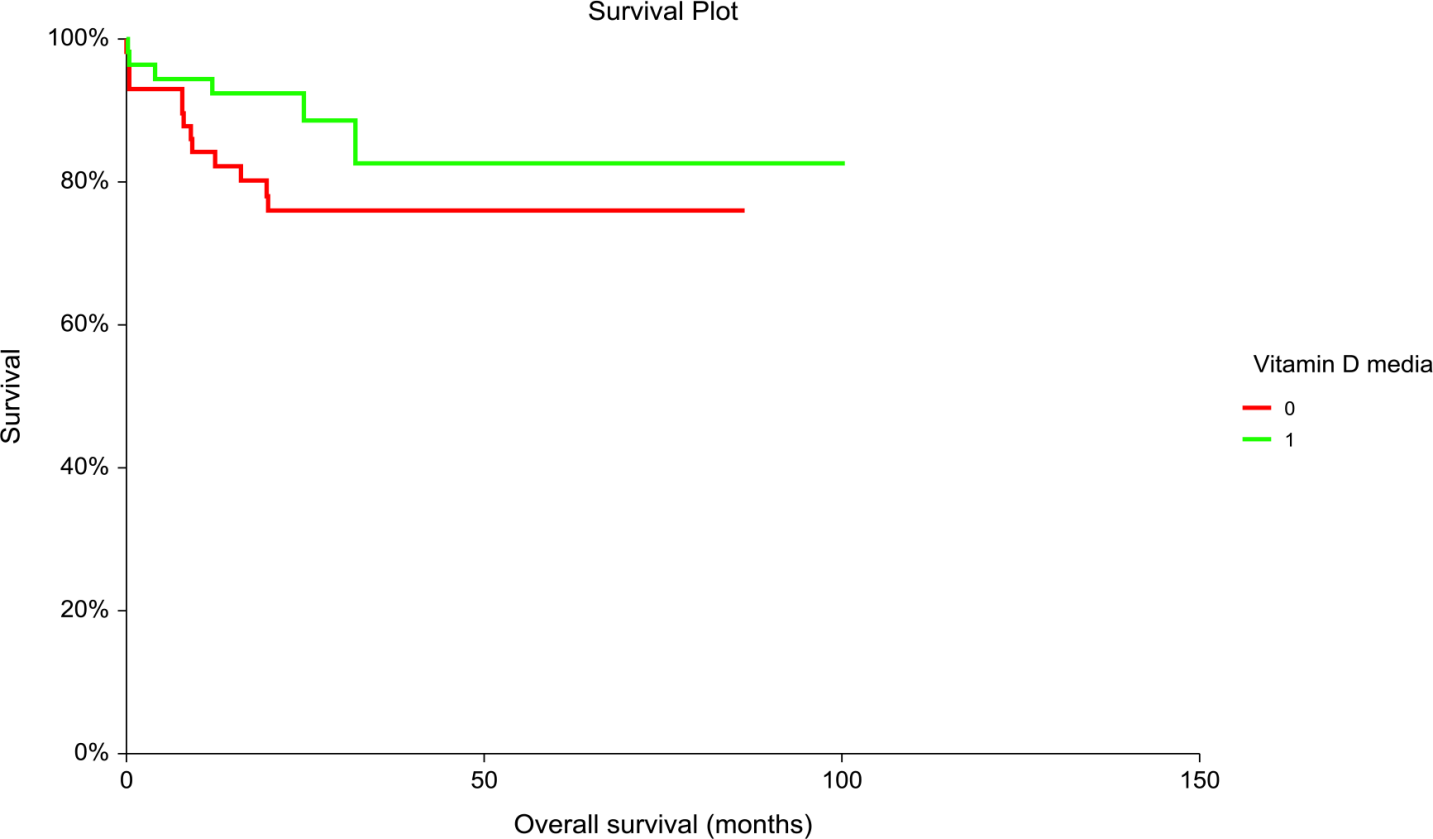
Kaplan–Meier estimates of probabilities of overall survival according to plasma vitamin D levels in TGCT patients (N = 120). Patients with plasma vitamin D concentration above median had non-significantly better OS as compared to patients with lower vitamin D concentration, [HR] = 2.06, 95% CI (0.84–5.06), p = 0.135; 0 = plasma vitamin D concentration below median, 1 = plasma vitamin D concentration above median). HR, hazard ratio; TCGT, testicular germ cell tumor; OS, overall survival; 0, plasma vitamin D concentration below median; 1, plasma vitamin D concentration above median.

Patients with “low” plasma vitamin D level had significantly inferior PFS but not OS compared to patients with “high” plasma vitamin D level (hazard ratio [HR] = 3.02, 95% CI (1.36–6.71), p = 0.01 for PFS and [HR] = 2.06, 95% CI (0.84–5.06), p = 0.14 for OS, respectively) (**Figures 1**, **2**). The prognostic impact of vitamin D level in PFS was observed in non-seminoma histology as well as in patients with clinical stages III.B–III.C. Metastatic disease (>3) retains prognostic significance, and metastatic sites such as liver metastases and retroperitoneal lymphadenopathy, as well as patients with non-pulmonary visceral metastases, were significantly associated with the prognostic impact between PFS and vitamin D plasma level (**Table 3**). Subgroup analysis revealed the prognostic value of plasma vitamin D level for OS and for PFS in patients with highly elevated serum tumor markers (S3); additionally, prognostic significance for OS was observed in patients with non-pulmonary visceral metastases as well as in a subgroup of patients with brain metastases. Vitamin D plasma level retains its prognostic significance in OS and in patients stratified according to treatment response, particularly in those who did not reach a complete response.

**Table 3:** Prognostic value of vitamin D.

	PFS				OS			
Variable	HR	95% CI low	95% CI high	P-value	HR	95% CI low	95% CI high	P-value
All patients	3.02	1.36	6.71	0.01	2.06	0.84	5.06	0.14
Histology								
Seminoma	0.00	0.00	0.00	0.09	0.00	0.00	0.00	0.17
Non-seminoma	2.58	1.10	6.07	0.04	1.83	0.71	4.74	0.23
Primary site								
testicle	2.91	1.31	6.79	0.02	1.95	0.79	4.8	0.17
extragonadal	0	0	0	1.0	0	0	0	1.0
Chemotherapy line								
1st line	1.88	0.73	4.86	0.20	1.53	0.52	4.54	0.44
>1st line	0	0	0	0.05	3.08	0.53	18.04	0.27
Stage of disease								
I.A–I.B stage	0	0	0	1.00	0	0	0	1.00
I.S stage	0	0	0	1.00	0	0	0	1.00
II.A–III.A stage	0	0	0	0.18	0	0	0	0.35
III.B–III.C stage	2.93	1.00	8.61	0.05	2.97	0.94	9.41	0.07
Relapsed	0	0	0	0.03	3.42	0.59	19.83	0.23
Serum tumor marker								
S0	0	0	0	0.33	0	0	0	1.00
S1	3.2	0.55	18.68	0.27	1.33	0.13	13.38	0.82
S2	2.31	0.4	13.17	0.29	2.32	0.41	13.28	0.29
S3	7.09	2.11	23.84	0.003	5.25	1.5	18.36	0.02
>S0	3.35	1.44	7.76	0.01	2.41	0.98	5.94	0.07
IGCCCG classification								
good	2.05	0.28	15.1	0.52	0.72	0.04	12	0.82
intermediate	3.29	0.32	33.99	0.30	1.94	0.25	15.4	0.50
poor	2.37	0.72	7.83	0.15	3.26	0.82	13.07	0.13
>good	2.69	0.92	7.83	0.06	2.58	0.83	8.09	0.11
Metastatic site								
Retroperitoneal metastases								
Present	3.61	1.58	8.21	0.004	2.45	0.99	6.03	0.06
absent	0	0	0	0.48	0	0	0	1.00
Medistinal Lymphadenopathy								
Present	3.49	0.8	15.2	0.21	2.12	0.37	12.21	0.48
Absent	2.35	0.88	6.27	0.10	1.65	0.56	4.91	0.37
lung metastases								
Present	5.07	1.83	14.05	0.004	3.04	0.98	9.44	0.08
Absent	1.83	0.49	6.76	0.38	1.21	0.27	5.32	0.80
Pulmonary metastases + lymph node except RP (M1a)								
Present	2.62	0.91	7.49	0.05	2.40	0.79	7.28	0.10
absent	0	0	0	0.17	0	0	0	0.36
Brain metastases								
Present	7.58	2.38	24.08	0.001	4.3	1.32	14.03	0.04
Absent	1.89	0.61	5.87	0.29	0.94	0.23	3.74	0.93
Liver metastases								
Present	–	–	–	–	–	–	–	–
Absent	2.47	1.03	5.94	0.05	1.5	0.54	4.11	0.44
Another site of metastases								
Present	0	0	0	0.38	0.65	0.05	8.14	0.68
Absent	2.43	1.01	5.83	0.06	1.78	0.62	5.07	0.29
Non-pulmonary visceral metastases (M1b)								
Present	4.5	1.3	15.59	0.03	7.05	1.9	26.11	0.03
absent	1.39	0.4	4.82	0.60	0.45	0.09	2.25	0.35
Lymphnode status								
N0–1	0	0	0	0.25	0	0	0	1
>N1	3.07	1.33	7.09	0.01	2.36	0.96	5.8	0.07
Another Lymphadenopathy								
Present	2.4	0.55	10.57	0.28	1.28	0.29	5.63	0.75
Absent	3.16	1.22	8.19	0.03	2.69	0.87	8.37	0.12
Number of metastatic sites								
0	0	0	0	0.48	0	0	0	1.00
1–2	0.70	0.14	3.52	0.67	0.31	0.05	1.79	0.26
>3	4.51	1.69	12.04	0.02	2.72	0.88	8.42	0.20
Response to Ctx								
Unfavorable respone								
Present	0	0	0	0.17	0.8	0.08	7.7	0.83
absent	1.05	0.3	3.61	0.94	0.53	0.11	2.63	0.45

PFS, progression free survival; OS, overall survival; CTx, chemotherapy; S, stage; IGCCCG, International Germ Cell Collaborative Cancer Group; N, node, CS, clinical stage; HR, hazard ratio; CI, confidence interval; PRm+, partial response marker positive; CR, complete response; PRm−, partial response marker negative.

We also looked for the kinetics of vitamin D levels between the 1st and 2nd cycles of chemotherapy, where patients with “low-low” compared to “high-high” had worse PFS (HR = 1.19, 95% CI 0.4–3.53, p = 0.03) and OS (HR = 1.05, 95% CI 0.38–2.96, p = 0.04).

## Discussion

In this translational study, we observed for the first time that vitamin D has a prognostic impact on patients with diagnosed germ cell tumors. The prognostic significance of vitamin D plasma level and progression-free survival was statistically significant among all studied patients; unfortunately, overall survival among all studied patients and its association with vitamin D plasma level failed to prove statistical significance. This observation is consistent with studies evaluating the prognostic impact of vitamin D in different types of cancer (41–43), whereas studies addressing the prognostic impact of vitamin D plasma level in testicular cancer are currently lacking. In our study group, the prognostic impact of vitamin D plasma level on overall survival was demonstrated in patients with highly elevated serum tumor markers (S3) and in patients with non-pulmonary and liver metastases. Thus, this prognostically significant observation in overall survival is mostly prominent in patients with advanced stages of disease. The prognostic impact of vitamin D in PFS, unlike in OS, among all studied patients might be explained by the fact that vitamin D levels might change over time during the course of the disease. Moreover, subsequent salvage treatment approaches might affect the prognostic value of vitamin D on OS as well. Interestingly, we also observed a significant association between vitamin D plasma levels and patient and tumor characteristics. Brain metastases as well as the number of metastatic sites (except for brain, liver, lung, and lymph nodes) were associated with a lower plasma vitamin D level. This observation is in concordance with studies (44, 45), which found a significant association between vitamin D plasma level and adverse clinico-pathological features in cancer, such as breast cancer or melanoma (44, 45). However, due to the limited number of patients with brain metastases, those results are hypothesis-generating and deserve further validation in a larger cohort of patients that includes patients with brain metastases. Those findings are also attributed to the fact that a low value of vitamin D is mostly associated with an advanced stage of disease in cancer (4, 45), which is in concordance with our study, where we observed a lower level of vitamin D in higher clinical stages of disease (III.B–III.C). Another significant fact observed among the studied group was that response to treatment in terms of partial response marker negative (PRm−) and complete response (CR) as well as patients without relapse were associated with higher plasma levels of vitamin D. Those observations raised several new questions.

Surprisingly, we did not observe a significant difference in vitamin D plasma level between GCT and healthy donors; moreover, we did not observe a significant difference in the month-matched analysis of vitamin D plasma value between HD and GCT patients. The mean vitamin D level among all studied patients was observed in the range of hypovitaminosis; thus, most patients were characterized by vitamin D insufficiency; likewise, the mean vitamin D level in HD was also in the range of hypovitaminosis (46). There is evidence that GCT survivors are associated with an insufficient level of vitamin D after treatment of primary cancer (47–50), with a significant difference compared to healthy donors (48, 49). An interesting study, which encompassed healthy donors as well as GCT patients, was outdone, where measurements of vitamin D values in GCT patients were assessed in longitudinal follow-up (>24 months) (51). In this study, decrement of vitamin D in GCT patients after orchiectomy was only transient, with a nadir at 6 months (mean), and thereafter, values comparable to healthy donors were observed (51). To sum it up, the mentioned studies proposed conflicting results: that vitamin D level in GCT patients and/or survivors is associated with hypovitaminosis, which could be transient, however, whether the potential explanation of hypovitaminosis might arise from its high prevalence in the general population is up for debate. Those conflicting results were also confirmed by a systematic review (52), where selection bias and different study designs could have an impact on the variability of results (52). Therefore, prospective studies to assess vitamin D variability and GCT patients according to treatment approaches and comparison with aged and month-matched blood draws from healthy donors are needed to establish its relationship. It is known that cholecalciferol is also produced in the skin and is mostly dependent on solar radiation (53), which is highest at latitudes below 40° (54). Moreover, dietary habits play an additional role as a source of vitamin D (55); thus, both factors may contribute to the high prevalence of vitamin D hypovitaminosis among populations in middle Europe, such as the Slovak population, which counts for more than 40% (56, 57). Therefore, we can conclude that GCT patients could be associated with an insufficient level of vitamin D after orchiectomy; however, a significant difference in vitamin D level between TGCT patients and the healthy population might not be observed, which is also in accordance with our results. Thus, hypovitaminosis in our cohorts might be due to the high prevalence of vitamin D insufficiency in the general population, which participated in our results. Moreover, we found out that a significant difference in vitamin D level was observed when poor responders were compared to HD. This observation could lead to the hypothesis that vitamin D might mirror a more aggressive disease.

We suggest that one possible explanation for why we observed a tendency of inverse association of vitamin D with brain metastases and the number of metastatic sites might arise from C-X-C motif chemokine ligand 12 and its receptor, C-X-C chemokine receptor type 4 (CXCL12-CXCR4), which are associated with the metastatic cascade (58). In a mouse breast cancer model, vitamin D deficiency increases lung expression of CXCL-12; therefore, CXCR-4-positive tumor cells follow the CXCL-12 gradient (36). Similar pathogenic pathways could run in GCT with brain metastases (59), with strengthened evidence, where the CXCL12-CXCR4 gradient was proposed as responsible for metastasis in the *in vitro* GCT model (60). Moreover, hypoxemia-induced factor 1-a (HIF-1a) is also affected by vitamin D, which plays a role in epithelial–mesenchymal transmission, migrations and has an impact on vascular proliferation (41); however, no study addressing the role of vitamin D and HIF-1a in germ cell tumors currently exists. Another significant role of vitamin D is the mediation of inhibition *via* the Wnt/β-catenin pathway (61), while it is already known that high β-catenin levels are associated with poor clinico-pathological features in germ cell tumors (62, 63). Another possible conjunction of vitamin D and progressive disease might be due to low expression of MAPKAPK2, which is already known to play a role in cancer invasion (64, 65). Higher MAPKAPK2 mRNA levels were observed in TGCT cell lines after vitamin D stimulation (23); thus, a potential low value of MAPKAPK2 due to vitamin D deficiency may enhance the activity of matrix metalloproteinase and its sequential invasion (25).

The possible explanation of the correlation between vitamin D and response to chemotherapy treatment in terms of PRm− or CR might be driven by study (66), where the authors conclude, that co-treatment of vitamin D and cisplatin on the germ cell line NTera2 in an *in vitro* model potentiated its effect. However, this observation was not proved by an *in vivo* model (66). Moreover, another mechanism is proposed by the Wnt/β-catenin pathway, which could contribute to cisplatin resistance (67–69), along with the fact that vitamin D is a Wnt/β-catenin pathway inhibitor (61). We could postulate hypothesis about why PRm− and CR were mostly seen among vitamin D proficient patients. However, we did not observe a significant relationship between vitamin D and cisplatin sensitivity among the studied group (p = 0.92); unfortunately, our cohort included only two patients with cisplatin-resistant disease, which may contribute to false negative results. The hypothesis that vitamin D could ameliorate cisplatin resistance in germ cell tumors is still up for research, while it is already known that vitamin D reduces cisplatin resistance in other cancers such as oral squamous cell carcinoma (70) and esophageal cancer (71). Therefore, we hypothesize that there might be an additional effect of vitamin D on cisplatin chemotherapy. Moreover, patients with low vitamin D values were characterized by higher relapse rates, suggesting that vitamin D deficiency is associated with disease and is more prone to relapse.

Numerous studies found a significant correlation between vitamin D value and overall and/or progression-free survival in cancers such as melanoma, colorectal, and breast (41–43). One possible explanation might be that vitamin D deficiency is associated with more aggressive elements in cancer, such as altered signaling pathways (1, 16, 23, 36), which may contribute to more unfavorable tumor phenotypes. However, a study evaluating whether vitamin D supplementation changes germ cell tumor patient survival has not yet been carried out; therefore, this question is still a challenge for researchers. While recent data from multi-analysis studies (72, 73) suggest a reduction in cancer mortality. Though various cancers were included in studies, drawing conclusions about their applicability to a precise type of malignancy from meta-analyses (72, 73) is questionable.

This study has several limitations. One of them could be the timeline of patient plasma sample storage, which for some patients is approximately 9 years. Another could be a small number of patients characterized by clinico-pathological findings, such as our cohort consisted mostly of patients in clinical stage III, whereas patients in clinical stage I were included in only 10%. Predominant (>70%) non-seminoma histology in our studied cohort is an additional study limitation that might have an impact on our results. Unlike seminoma, non-seminoma histology might be categorized as a poor-risk disease, and its adverse tumor and/or clinical features are associated with vitamin D deficiency; hence, hypovitaminosis prevalence and impact on PFS among the studied cohort might be affected by a significant contribution from non-seminoma histology predominance with higher clinical stage in our studied cohort. Confounding factors could play an important role in this study, such as patient sun exposure during the season. Most of our blood samples were collected during the winter season (December, January, February, and March) compared to the summer season (June, July, August, and September), which counts for 40% vs 28% among the studied cohort, respectively. Vitamin D level was also compared according to quarter of year, where highest level of vitamin D was observed during summer quarter. This general fact might be associated with higher sun exposure during summer. Thus, extensive, or low sun exposure depending on the season may affect vitamin D plasma values. The performance status of patients in advanced stages of disease must be considered as well. Patients with more advanced stages of disease tend to present with worsening symptoms such as fatigue, weakness, pain, and decreased social interaction (74, 75), which may have an impact on time spent outdoors.

In conclusion, this is the first report that proves an association between GCT patients and clinico-pathological findings; furthermore, we report a significant correlation between plasma vitamin D level and overall and/or progression-free survival in selected populations. This conclusion opens up the question of whether vitamin D supplementation proves advantageous in OS or PFS in a precisely selected population. Those questions deserve further evaluation in animal and interventional studies. Moreover, our observation suggests that a low vitamin D level is associated with relapse in GCT. This observation deserves further evaluation and should be applied and re-evaluated in a more selected population, such as a stage I germ cell tumor, which could provide additional insight into stratifying patients.

## Data availability statement

The raw data supporting the conclusions of this article will be made available by the authors, without undue reservation.

## Ethics statement

The studies involving human participants were reviewed and approved by Institutional review Board of National Cancer institute of Slovakia. The patients/participants provided their written informed consent to participate in this study.

## Author contributions

PL, MM, and KK wrote the main manuscript text, analyzed, and discussed the data. KK, PC, and BV prepared samples and performed experiments. PL, KK, PC, and MM contributed to the interpretation of the results, revised, and edited the manuscript. MM made substantial contributions to the conception and design of the work, revised, and edited the manuscript. All authors listed have made a substantial, direct, and intellectual contribution to the work and approved it for publication.
